# The microRNAs landscape of luminal B breast cancer cells in a three-dimensional microenvironment

**DOI:** 10.1186/s41065-025-00586-2

**Published:** 2025-10-29

**Authors:** Stephanie I. Nuñez-Olvera, Lorena Aguilar-Arnal, Jonathan Puente-Rivera, Alfredo Hidalgo-Miranda, Mireya Cisneros-Villanueva, Yarely M. Salinas-Vera, Laurence A. Marchat, María Elizbeth Álvarez-Sánchez, Karla Rubio, César López-Camarillo

**Affiliations:** 1 Departamento de Biología Celular y Fisiología, Instituto de Investigaciones Biomédicas, Universidad Nacional Autónoma de México, Ciudad de México, 04510 México; 2 División de Investigación, Hospital Juárez De México, Ciudad de México, 07760 México; 3 Laboratorio Genómica del Cáncer, Instituto Nacional de Medicina Genómica, Ciudad de México, 14610 México; 4 Posgrado en Ciencias Genómicas, Universidad Autónoma de la Ciudad de México, Ciudad de México, 03100 México; 5 Programa Institucional de Biomedicina Molecular, Escuela Nacional de Medicina y Homeopatía del Instituto Politécnico Nacional, Ciudad de México, 07320 México; 6 Laboratorio Internacional EPIGEN, Consejo de Ciencia y Tecnología del Estado de Puebla (CONCYTEP), Instituto de Ciencias, Benemérita Universidad Autónoma de Puebla, EcoCampus, Puebla 72570 México

**Keywords:** Breast cancer, 3D cultures, MicroRNAs, MiRNA/lncRNAs/mRNA coregulation networks

## Abstract

**Background:**

Three-dimensional (3D) culture captures key features of tumor architecture and microenvironmental signaling. Because microRNAs (miRNAs) are central post-transcriptional regulators, we asked whether 3D growth would reveal disease-relevant lncRNA-miRNA-mRNA programs in BT-474 luminal breast cancer cells.

**Methods:**

BT-474 cells were profiled with GeneChip miRNA 4.0 microarrays to compare 3D versus 2D conditions. We then performed an integrative analysis consisting of: (i) extracting miRNA–mRNA interactions from miRNet and ENCORI (predicted and curated) for the differentially expressed miRNAs; (ii) identifying inverse miRNA–mRNA pairs (miRNA downregulated with target mRNA upregulated, or the reverse) using public mRNA data from BT-474 3D versus 2D cultures so that pairs were consistent with regulatory repression; and (iii) retaining only those miRNA–mRNA pairs that also appeared in miRNet/ENCORI. In a separate step, we examined public luminal breast tumor datasets versus BT-474 2D cultures to determine which 3D-identified miRNAs showed a similar direction of expression change in luminal tumors, thereby prioritizing signals with putative in vivo relevance. Finally, to enhance biological plausibility, we incorporated an lncRNA layer by leveraging their miRNA “sponge” (ceRNA) activity, and we assembled an integrated miRNA–mRNA–lncRNA network. Key miRNAs from this network were then evaluated for expression in external TCGA cohorts and for diagnostic potential using area-under-the-curve (AUC) analysis.

**Results:**

Our data reveal a reprogramming of miRNA expression between 3D and 2D cultures. Using a threshold of FC > 1.5 and *p* < 0.05, we found 75 miRNAs upregulated and 82 downregulated in 3D relative to 2D. To strengthen biological interpretation, we integrated our miRNA expression data with public mRNA profiles from BT-474 cells cultured in 3D versus 2D. We then cross-referenced interactions reported in miRNet and ENCORI, retaining only miRNA–mRNA pairs showing inverse regulation. This integrative layer yielded a pathway-level view in which 3D-modulated miRNAs collectively favored proliferative programs, ESR1/estrogen signaling, MAPK/JNK activity, cell-cycle control, and extracellular-matrix remodeling, with relative attenuation of EGFR/TGF-β signaling. Together, the inferred miRNA–mRNA network supports a shift toward a more tumor-like, transcriptionally active state in 3D. To reinforce translational relevance, we cross-referenced public luminal breast tumor datasets with BT-474 2D cultures and identified 14 miRNAs in common, with the same direction of change as observed in 3D cultures. From these, we assembled co-regulatory networks linking miRNAs to their mRNA targets and to lncRNAs. This analysis highlighted four key miRNA nodes: miR-92a-3p, miR-539-5p, miR-18a-5p, and miR-130a-3p, which are connected to 58 mRNAs and modulated by 12 lncRNAs. The hubs converge on pathways for growth and survival via the RTK–MAPK axis, estrogen signaling, and fibronectin/adhesion–mechanotransduction processes, depicting a proliferative, mechano-responsive phenotype. Additionally, these miRNAs emerged as potential biomarker candidates: in TCGA datasets, miR-539-5p, miR-18a-5p, and miR-130a-3p are repressed in luminal breast tumors compared to normal breast tissue, each showing moderate to good diagnostic performance (AUC ~ 0.70–0.80).

**Conclusion:**

Overall, this study highlights the utility of 3D cultures in investigating the biology of breast cancer, emphasizing the critical interplay between lncRNAs, miRNAs, and mRNAs. Our findings provide insights into the discovery of novel potential biomarkers, underscoring the importance of integrating both in vitro 3D culture and in vivo expression data for a more accurate identification of miRNAs with possible applications in cancer therapies.

**Supplementary Information:**

The online version contains supplementary material available at 10.1186/s41065-025-00586-2.

## Introduction

Breast cancer remains one of the most prevalent and lethal cancers worldwide, with progression driven by alterations in gene expression [1, 2]. Among breast cancer subtypes, luminal B comprises ER-positive tumors with low progesterone-receptor expression, high proliferation, and poorer endocrine responses than luminal A [3, 4], with outcomes comparable to HER2-enriched subtype. BT-474, which co-expresses ER, PR, and HER2, is widely used as a luminal B model and provides a relevant platform for experimental studies [5]. In addition, luminal B tumors have been poorly studied, representing 11.2% of cases relative to the luminal A subtype (72.6%) [6]. These clinicopathologic features reflect dysregulated gene expression, in which non-coding RNAs, including microRNAs (miRNAs) and long non-coding RNAs (lncRNAs), are key determinants of tumor phenotypes [7]. miRNAs, small RNA molecules of approximately 21–24 nucleotides, primarily function by binding to the 3′ untranslated regions (3′UTR) of target mRNAs, leading to post-transcriptional repression, degradation of mRNA, or inhibition of translation [8]. These small RNAs are involved in several essential cellular processes, including proliferation, differentiation, apoptosis, and development, all closely linked to cancer progression [9–14]. On the other hand, lncRNAs have emerged as crucial players in cancer biology. Abnormal expression of lncRNAs has been associated with various human diseases, including cancer [15–17]. These molecules exert their influence by interacting with miRNAs, mRNAs, and even chromatin, affecting the expression of genes involved in cellular processes such as tumorigenesis, metastasis, and drug resistance [10, 18, 19]. Several studies have identified specific miRNAs and lncRNAs that are differentially expressed in breast cancer, suggesting their potential as biomarkers and therapeutic targets for the disease [20–22]. Mechanisms by which miRNAs and lncRNAs exert their regulatory effects are through the competing endogenous RNA (ceRNA) network. This regulatory system suggests that lncRNAs can act as natural miRNA sponges, sequester miRNAs, and prevent them from binding to their target mRNAs [23]. Most ceRNA studies in cancer have been conducted using traditional two-dimensional (2D) cell culture models [24–26]. For example, in MCF-7 cells, integrated miRNA–mRNA–lncRNA analysis identified a miR-19a–(ESR1/FMNL2/CD74)–DLEU1 axis that potentially modulates metastasis and immunity-related pathways in breast cancer [27]. Additionally, ceRNA networks reported in tumor-derived exosomes from luminal B breast cancer, comprising 16 lncRNAs, 15 miRNAs, and 15 mRNAs, primarily modulate RAS–MAPK signaling and regulation of the MAPK cascade, with specific axes such as lncRNA OAZ1–miR-7851-3p–ALG12/HOXA5 that may contribute to this subtype’s biology and have prognostic relevance [28]. Given the distinctive features of three-dimensional (3D) culture, elucidating ceRNA networks under these conditions can reveal context-dependent interactions shaped by extracellular-matrix architecture, cell–cell communication, and mechanical cues, thereby prioritizing regulatory axes likely operative in tumors and highlighting candidates with translational potential. In our work, we uncover broad miRNA reprogramming between 3D and 2D culture: 75 upregulated and 82 downregulated in 3D (FC >1.5; *p* < 0.05). Integrating our miRNA expression analysis with public BT-474 mRNA data, we find miRNA–mRNA pairs that converge on ESR1/estrogen, MAPK/JNK, cell-cycle control, and ECM remodeling, with relative attenuation of EGFR/TGF-β pathways. The network indicates a more tumor-like, transcriptionally active 3D state. Cross-checks with luminal tumor datasets show that 14 miRNAs are shared between 3D cultures and luminal tumors, among which four (miR-92a-3p, miR-539-5p, miR-18a-5p, miR-130a-3p) link to 58 mRNAs and 12 lncRNAs. These miRNAs also show promise as potential biomarkers: in TCGA, miR-539-5p, miR-18a-5p, and miR-130a-3p are downregulated in luminal breast tumors relative to normal tissue, each achieving moderate-to-good discrimination (AUC ≈ 0.70–0.80). This comprehensive network provides valuable insights into the molecular mechanisms underlying breast cancer progression and may serve as a foundation for identifying novel therapeutic targets. The findings could pave the way for more effective preclinical models and better therapeutic strategies for breast cancer.

## Materials and methods

### Generation of 3D cultures from BT-474 cells

To establish the 3D cultures, we sourced BT-474 cell lines from ATCC (Manassas, Virginia) and cultured them in HybriCare medium supplemented with 10% fetal bovine serum (FBS). In 24-well plates, we added 120 µL of Geltrex (Thermo Fisher Scientific) and allowed it to solidify at 37 °C for 30 min. Subsequently, we introduced 3.1 × 10⁴ cells from the 2D BT-474 culture and 250 µL of HybriCare medium and incubated them at 37 °C for an additional 30 min. Finally, we added 250 µL of HybriCare medium mixed with 5% Matrigel. Over the following days, the 3D cultures were refreshed with 500 µL of complete medium every three days.

### RNA isolation from BT-474 cells in 2D monolayer and 3D cultures

To analyze miRNA expression, total RNA was extracted from BT-474 cells cultured in both 2D monolayer and 3D cultures. For this, we started with 3.1 × 10⁴ cells, which were incubated for 6 days. We added 1 mL of Trizol to the 2D and 3D cultured cells and mixed them by pipetting. After allowing the cell lysate to incubate at room temperature for 5 min, 200 µL of chloroform per mL of Trizol reagent was added and the tube was vigorously shaken for 15 s. The tube was then incubated at room temperature for 3 min before being centrifuged at 12,500 rpm for 25 min at 4 °C. After centrifugation, we carefully collected the aqueous phase without disturbing the other layers and transferred it to a new Eppendorf tube. 500 µL of isopropanol was added to precipitate the RNA, and the pellet was incubated on ice for 20 min. The tubes were then centrifuged at 12,500 rpm for 25 min at 4 °C. After discarding the supernatant, we washed the pellet with 1 mL of 75% ethanol and allowed it to air-dry for 5 min. Finally, the pellet was resuspended in 20 µL of nuclease-free water. The extracted RNA was quantified using spectrophotometry and visualized by 1% agarose/TAE 1× electrophoresis.

### Microarray assays

We employed the GeneChip miRNA Array 4.0 (Affymetrix GeneChip, Santa Clara, CA, USA) to analyze miRNA expression profiles with biological triplicates for 2D and 3D culture conditions (*n* = 3). Following the manufacturer’s protocol, we used 100 ng of total RNA to synthesize the first-strand cDNA. RNA was degraded, and second-strand cDNA was synthesized using DNA polymerase I and RNase H. The sense-strand cDNA was subsequently synthesized and used for microarray experiments. A key step, reverse transcription, was carried out to generate the sense-strand cDNA from the RNA samples. This cDNA was loaded onto the GeneChip Array provided by Thermo Fisher Scientific (Waltham, MA, USA). The arrays were incubated at a constant temperature of 45 °C for 16 h in the GeneChip Hybridization Oven 645 (Affymetrix Inc., Santa Clara, CA, USA). Following the incubation, hybridization was performed using the GeneChip Fluidics Station 450. We scanned the arrays using the GeneChip Scanner 3000 7G (Affymetrix Inc., Santa Clara, CA, USA) to analyze gene expression profiles. Raw.CEL files were imported into Transcriptome Analysis Console (TAC; Thermo Fisher Scientific, v [4.x]). Array quality was assessed with TAC’s QC panel (intensity distributions, array-to-array correlations, PCA). Data were normalized using SST-RMA (Signal Space Transformation–RMA) with background correction, quantile normalization, and probe-set summarization. Differential expression (3D vs. 2D) was computed on the log₂ SST-RMA values using TAC’s limma-based statistical framework (moderated t/empirical Bayes). For each feature we report log₂ fold change (log₂FC) as the difference of group means; linear FC was obtained as log₂FC. *P*-values were adjusted for multiple testing by Benjamini–Hochberg FDR; significance thresholds (FDR < 0.05 and log₂FC ≥ 1).

### Publicly available microarray data and analysis

Public data for mRNAs in BT-474 3D cultures (GSE206836), miRNAs in luminal breast cancer tumors (GSE225292), and miRNAs from 2D cultures of BT-474 cells (GSE163490) were collected from the Gene Expression Omnibus (GEO) database. We analyzed miRNAs from luminal breast cancer tumors and 2D cultures of BT-474 cells using the authors’ normalized log₂ expression provided in the GEO Series Matrix files: luminal breast tumors (platform GPL18402) and BT-474 cells grown in 2D (platform GPL16770). To avoid cross-platform artifacts, the two cohorts were analyzed individually on their native Agilent log₂ scales with no cross-platform merging or additional normalization/batch correction. miRNAs were matched by standard miRNA name. We did not re-annotate probes or collapse probe signals; each row corresponds to the miRNA as reported in the respective matrix. For limits of detection and inclusion criteria, we use Agilent’s sentinel value − 9.969 (“not detected”) was handled as missing/off-scale in the primary analysis, and we restricted interpretation to miRNAs with RMA >1 across samples. For each miRNA, we computed log2FC = log2(tumor) − log2(BT-474), and group differences were tested on the log₂ scale using a two-sided Welch’s t-test (full per-miRNA outputs in Supplementary Data S4). For differential expression of mRNA in 3D vs. 2D culture, the analysis steps were analogous: CEL files were imported into TAC, v4.x, normalized with SST-RMA, and differential expression between 3D and 2D was assessed on the log₂ scale using TAC’s limma-based framework (moderated t/empirical Bayes). We then performed a biological integration using miRNet and ENCORI databases [26, 27]. Specifically, results were matched only at the interaction level, retaining pairs where (i) the miRNA was down-regulated (3D vs. 2D), (ii) its mRNA target was up-regulated (3D vs. 2D), and (iii) the pair appeared in miRNet or ENCORI (predicted/curated evidence). This strategy focuses on the directional concordance expected for regulatory pairs (miRNA↓/target mRNA↑). Furthermore, we assessed the performance of these miRNAs using a metric called the area under the curve (AUC) values to predict if miRNA expression could discriminate between normal and breast cancer samples. The AUC values were retrieved from CancerMIRNome [29], and the TCGA expression analyses were obtained from the UALCAN database [30].

### Statistical analyses

All statistical analyses were performed using GraphPad Prism version 8. Data are presented as mean ± standard error of the mean (SEM) unless otherwise indicated. For experiments involving more than two groups, one-way or two-way analysis of variance (ANOVA) was used, followed by Tukey’s post hoc test, to assess group differences. *p ≤ 0.05, **p ≤ 0.01, ***p ≤ 0.001.

## Results

### Morphological characterization and microRNAs gene expression in organotypic 3D cell cultures

**Fig. 1:**
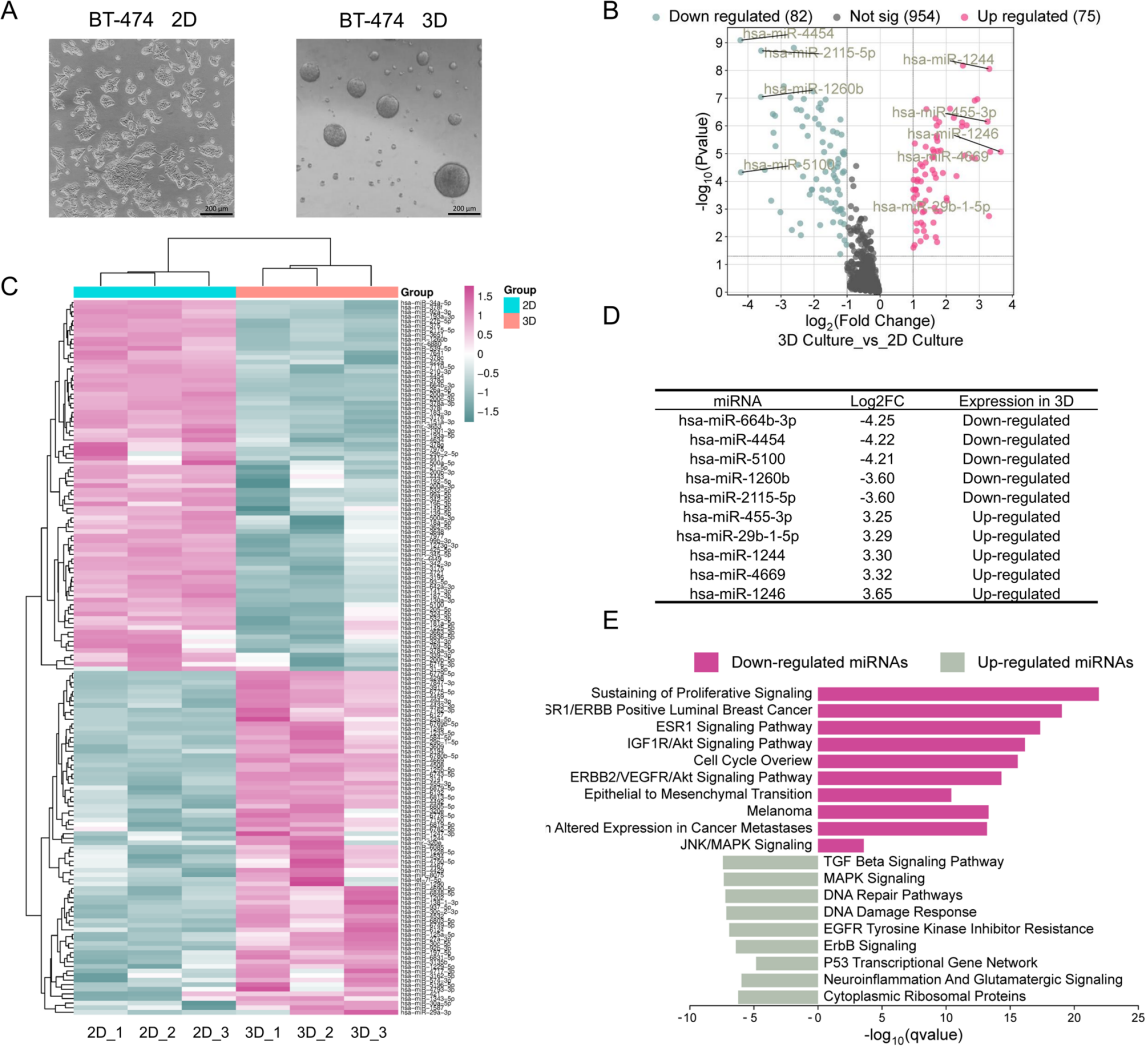
Expression profile of miRNAs in 2D monolayers and 3D organotypic cell cultures. **A** Representative optical microscopy images comparing the morphological differences between 2D cultured cells (left panel) and 3D structures (right panel) of BT-474 cells. **B** Volcano plot depicting the distribution of miRNAs based on fold change and statistical significance. Upregulated miRNAs in 3D cultures are shown in pink dots, downregulated in green dots, and non-significant changes in black. Selected deregulated miRNAs with the most significant fold changes are indicated. *P*-values were adjusted for multiple testing by Benjamini–Hochberg FDR; significance thresholds (FDR < 0.05 and FC > 1.5) (**C**) Heatmap showing hierarchical clustering of differentially expressed miRNAs between 2D and 3D culture conditions. Each row represents a specific miRNA with color intensity indicating relative expression levels (pink: upregulated, green: downregulated). Heatmap constructed from the RMA-normalized expression matrix (log2); hierarchical clustering was performed using Euclidean distance. **D** Table summarizing fold change values and expression status of the top ten differentially expressed miRNAs in 3D cultures. **E** Enriched pathways for downregulated (pink bars) and upregulated (gray bars) miRNAs in 3D cultures, showing -log10 q-values for each path. Pathways related to proliferation, cell cycle, and cancer metastasis are enriched among downregulated miRNAs, whereas pathways associated with DNA damage response and apoptosis are enriched among upregulated miRNAs

To explore the impact of 3D culturing on BT-474 luminal B breast cancer cells morphology, we conducted a characterization using optical microscopy to examine the cellular architecture. Cells cultured in 3D formed compact spheroids with smooth surfaces and a homogeneous, mass-like morphology that obscured individual cell boundaries (Fig. 1A, right panel). In contrast, cells grown under traditional 2D monolayer conditions exhibited distinct, patchy arrangements with clearly defined cellular contours (Fig. 1A, left panel). These structural differences highlight the profound changes in cell organization and microenvironmental interactions that occur when cells are cultured in 3D. Based on these morphological findings, we then examined whether the 3D culture environment also affected the regulation of gene expression at the post-transcriptional level. To achieve this, we conducted a comparative analysis of global miRNA expression using the GeneChip miRNA 4.0 microarray platform, which includes 5,214 probes targeting mature human miRNAs. Our results revealed a substantial alteration in miRNA expression when cells were cultured in 3D compared to conventional 2D conditions. Specifically, 75 miRNAs were significantly upregulated, while 82 miRNAs were notably downregulated in 3D cultures (fold change > 1.5, adjusted *p*-value < 0.05) (Fig. 1B and Supplementary data). These differentially expressed miRNAs segregated into two distinct clusters as illustrated in the heatmap (Fig. 1C). The inset table in Fig. 1D lists the ten most prominently downregulated miRNAs in 3D conditions, including hsa-miR-664b-3p, hsa-miR-4454, hsa-miR-5100, hsa-miR-1260b, and hsa-miR-2115-5p. The top upregulated miRNAs comprised hsa-miR-455-3p, hsa-miR-29b-1-5p, hsa-miR-1244, hsa-miR-4669, and hsa-miR-1246. Functional pathway enrichment analysis indicates that the downregulation of these miRNAs likely leads to the activation of critical signaling pathways associated with sustaining proliferative signaling, estrogen receptor (ESR1)/ERBB-positive luminal breast cancer, ESR1 signaling, and JNK/MAPK signaling (Fig. 1E, upper panel). This observation aligns with previous reports indicating enhanced activation of the JNK/MAPK pathways in 3D cultures of breast cancer cell lines, such as T47D, suggesting that miRNA downregulation may facilitate these proliferative and survival signaling mechanisms during 3D growth. On the other hand, the upregulated miRNAs in 3D cultures appear to target tumor suppressor pathways, including the TGF-β, MAPK signaling, and TP53 signaling pathways (Fig. 1E, lower panel).

### Enrichment of key signaling pathways in 3D cultures of BT-474 cells

**Fig. 2:**
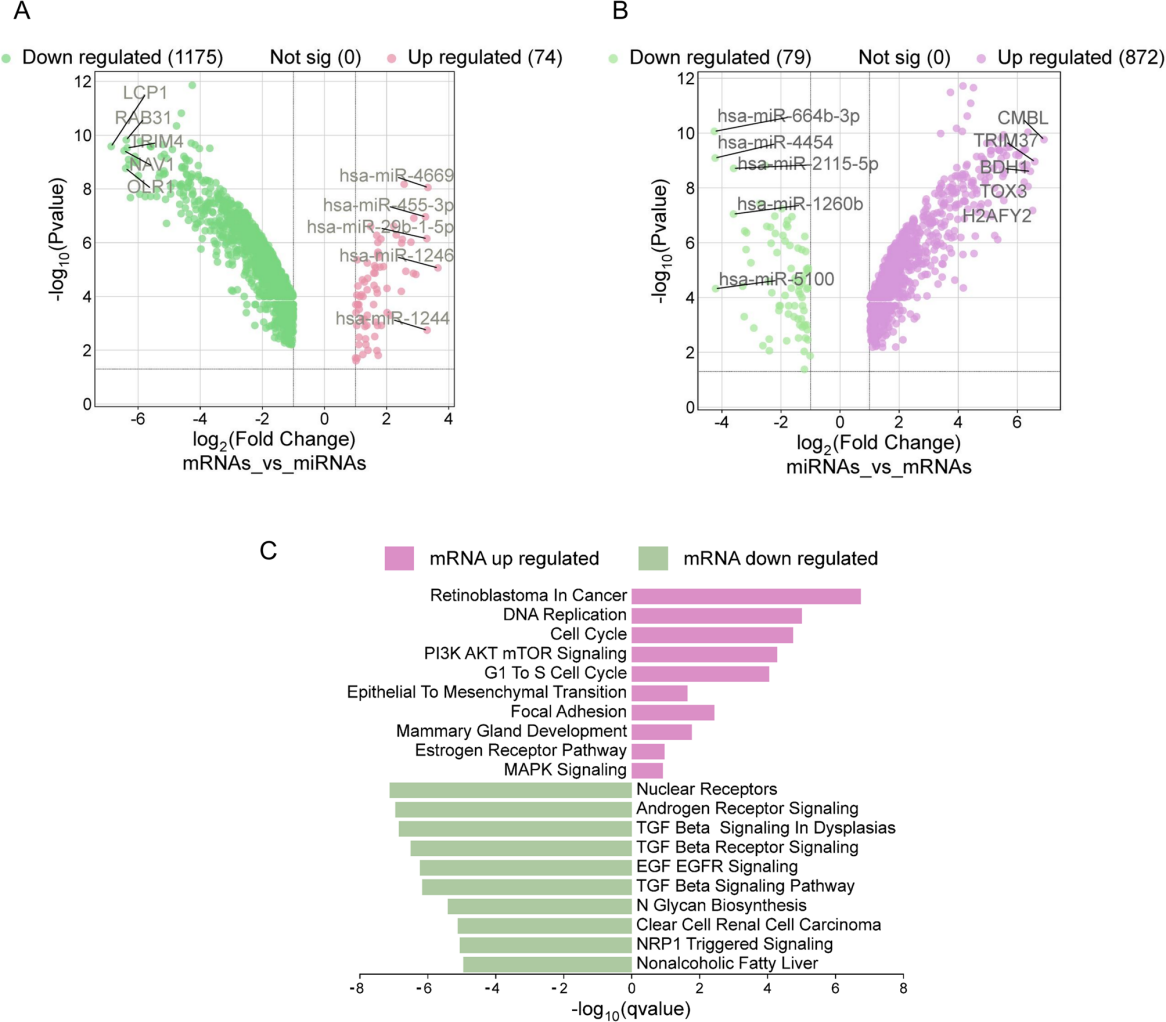
Analysis of mRNA expression and miRNA-mRNA interactions in 3D cultures of BT-474 cells. **A** Volcano plot of mRNAs downregulated in 3D cultures that are targets of miRNAs upregulated in 3D. *P* values were adjusted for multiple testing using the Benjamini–Hochberg method; significance was defined as FDR < 0.05 and FC > 1.5. **B** Volcano plot of mRNAs upregulated in 3D cultures that are targets of miRNAs downregulated in 3D. *P* values were adjusted for multiple testing using the Benjamini–Hochberg method; significance was defined as FDR < 0.05 and FC > 1.5 (**C**) Enrichment analysis of biological processes associated with 872 upregulated mRNAs—predicted targets of 79 downregulated miRNAs—and 1,175 downregulated mRNAs—predicted targets of 74 up-regulated miRNAs. Upregulated mRNAs (pink bars) Downregulated mRNAs (green bars). The figure shows –log₁₀ q-values for each pathway as a measure of statistical significance

To provide a more biologically relevant context to the enriched processes, we integrated publicly available mRNA expression data from 3D cultures of BT-474 cells (GSE206836), which we previously reported [31]. Specifically, the mRNA targets of the differentially expressed miRNAs were predicted using Encori and miRNET software and compared with the mRNA expression dataset obtained from 3D cultures. We focused on miRNA-mRNA pairs where the fold change values were in opposite directions. This analysis revealed 1,175 mRNAs were downregulated in 3D cultures, which were predicted to be targets of 74 upregulated miRNAs (Fig. 2A and Supplementary data S3). On the other hand, 872 mRNAs upregulated in BT-474 3D cell cultures and predicted to be targets of 79 downregulated miRNAs mapped here (Fig. 2B and Supplementary data S2). Subsequently, we used these mRNA–miRNA pairs to perform an enrichment analysis to identify the associated biological processes. The set of 74 upregulated miRNAs was significantly enriched in pathways related to TGF-β signaling and G1/S DNA damage checkpoint regulation (Fig. 2C). In contrast, 872 upregulated mRNAs—predicted targets of 79 downregulated miRNAs—were associated with cell cycle progression, DNA replication, and focal adhesion pathways. Notably, we observed enrichment in signaling pathways such as MAPK and AKT signaling, and ESR1-mediated signaling, as well as biological processes related to epithelial-to-mesenchymal transition (Fig. 2C). We found a striking consistency when comparing these results with the predictive pathway analysis. Both analyses revealed enrichment in the estrogen-related ESR1 and the MAPK/JNK signaling pathways, further strengthening the hypothesis that these pathways play a key role in the biological processes associated with 3D cell culture. The convergence of these findings highlights the robustness of ESR1 and MAPK/JNK signaling as central processes in the 3D culture system, providing more substantial evidence that these pathways are critical in modulating the behavior of breast cancer cells in three-dimensional environments.

### Exploring shared MiRNAs in 3D cultures and luminal breast cancer tumors

**Fig. 3:**
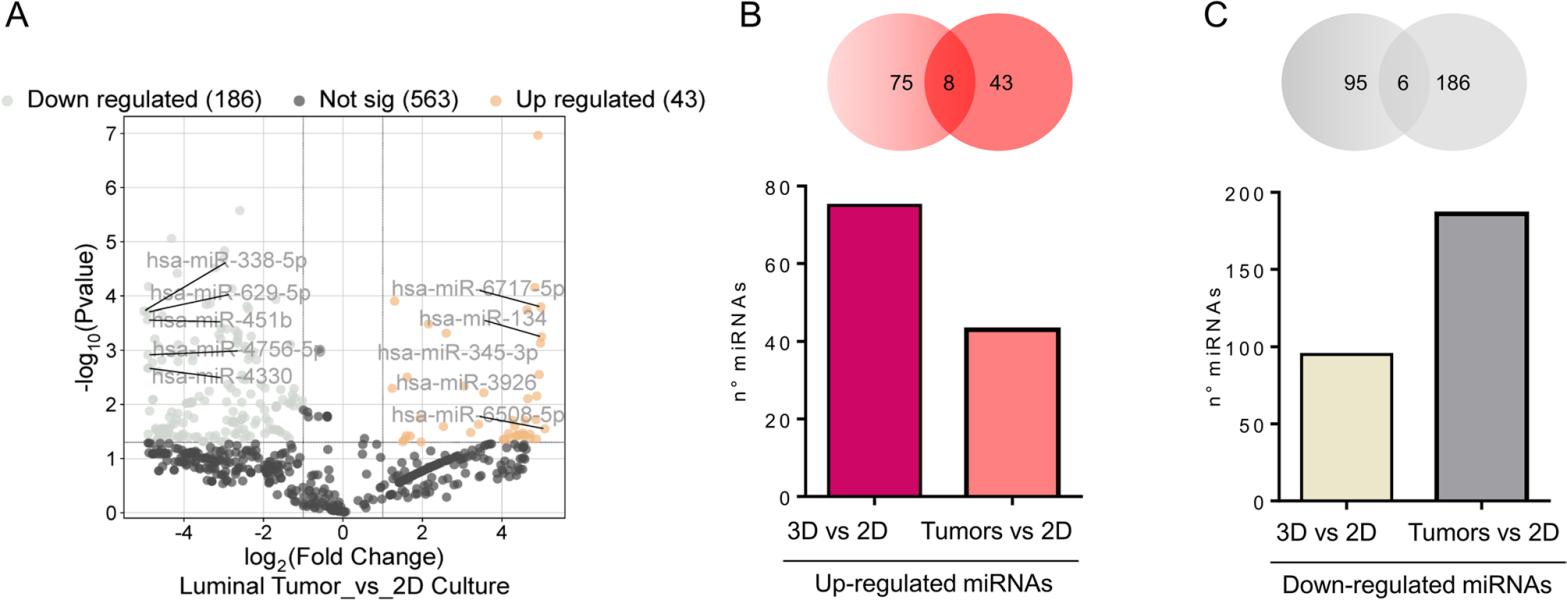
Integrating miRNA expression profiles from 3D and 2D cultures of BT-474 cells and luminal breast tumors. **A** Volcano plot displaying the differential expression of miRNAs in tumors versus 2D cultures of BT-474 cells, highlighting upregulated (red) and downregulated (gray) miRNAs. For each miRNA, log₂FC = mean(tumor) − mean(BT-474) (linear FC = 2^ [32]); group differences were tested on the log₂ scale using two-sided Welch’s t-tests. **B** Venn diagram shows the overlap between miRNAs upregulated in 3D vs. 2D cultures and those upregulated in luminal breast tumors. The intersection highlights miRNAs upregulated in both contexts (shared by tumor and 3D culture). **C** Venn diagram shows the overlap between miRNAs down-regulated in 3D vs. 2D cultures and those down-regulated in luminal breast tumors. The intersection highlights miRNAs in both contexts (shared by tumor and 3D culture)

Integrating data from tumor samples significantly may enhance experimental findings’ robustness and biological relevance. In this analysis, we compared miRNA expression profiles from two distinct sources: (i) the dataset generated in this study, which includes miRNAs differentially expressed between 3D and 2D cultures of BT-474 cells, and (ii) publicly available miRNA data from luminal breast tumors (GSE225292) and 2D cultures of BT-474 cells (GSE163490). By cross-referencing miRNAs with differential expression across these datasets, we aimed to identify miRNAs consistently regulated across distinct tumor microenvironments, particularly those showing similar expression in 3D cultures and luminal breast tumors, which could help reduce the gap between in vitro and in vivo data. Our analysis revealed that, in luminal breast cancer tumors, 43 miRNAs were upregulated compared to the 2D culture, and 186 miRNAs were downregulated (FC ≥ 1.5 and a *p*-value < 0.05) (Fig. 3A and Supplementary data S4). Following this, we conducted an overlapping miRNA analysis, identifying 8 upregulated miRNAs common to both the 3D culture and luminal tumor samples, compared to the 2D culture system. Additionally, we identified 6 downregulated miRNAs that were shared between the 3D cultures and luminal tumors (Fig. 3, panels B-C). Table 1 summarizes the fold change values of the identified shared miRNAs, their established biological functions, and experimentally confirmed targets. This approach strengthens confidence that these miRNAs play a pivotal role in cancer biology, rather than coincidental findings specific to a single model. Identifying overlapping miRNAs between these different contexts—3D cultures, 2D cultures, and luminal tumor samples—emphasizes the relevance of these molecules as potential biomarkers for breast cancer.

**Table 1:** Shared MiRNAs in 3D models and luminal B breast cancer tissues versus conventional 2D BT-474 cultures

miRNA	Log2FC 3D vs. 2D	Log2FC Tumors vs. 2D	Function	Target	Reference
hsa-miR-92a-3p	−3.49	−4.27	Cell proliferation and metastasis	BTG2	[33]
hsa-miR-378d	−2.52	−3.35	Inhibits Growth and Migration	XBP1	[32]
hsa-miR-130a-3p	−2.41	−3.71	Inhibits cell proliferation	RAB5B	[34]
hsa-miR-362-5p	−2.07	−2.12	Cell proliferation and migration	CYLD	[35]
hsa-miR-18a-5p	−1.13	−1.97	Inhibits migration and adherence	PI3K/P-AKT	[36]
hsa-miR-539-3p	−1.11	−4.58	Inhibits proliferation and migration	EGFR	[37]
hsa-miR-4717-3p	1.10	1.41	Cell proliferation and growth	METTL3	[38]
hsa-miR-5196-5p	1.36	2.60	Uknown	Uknown	NA
hsa-miR-4534	1.37	1.86	Cell proliferation and migration	PTEN	[39]
hsa-miR-1290	1.73	2.15	Cell viability and proliferation	NLRP3	[40]
hsa-miR-3141	1.73	2.15	Uknown	Uknown	NA
hsa-miR-4433-3p	1.73	4.82	Uknown	Uknown	NA
hsa-miR-3911	1.89	3.87	Metastasis	SLIT2	[41]
hsa-miR-584-5p	2.78	4.35	Migration and invasion	MSMO1	[42]

### Exploring CeRNA networks in 3D cultures and luminal breast cancer tumors

**Fig. 4:**
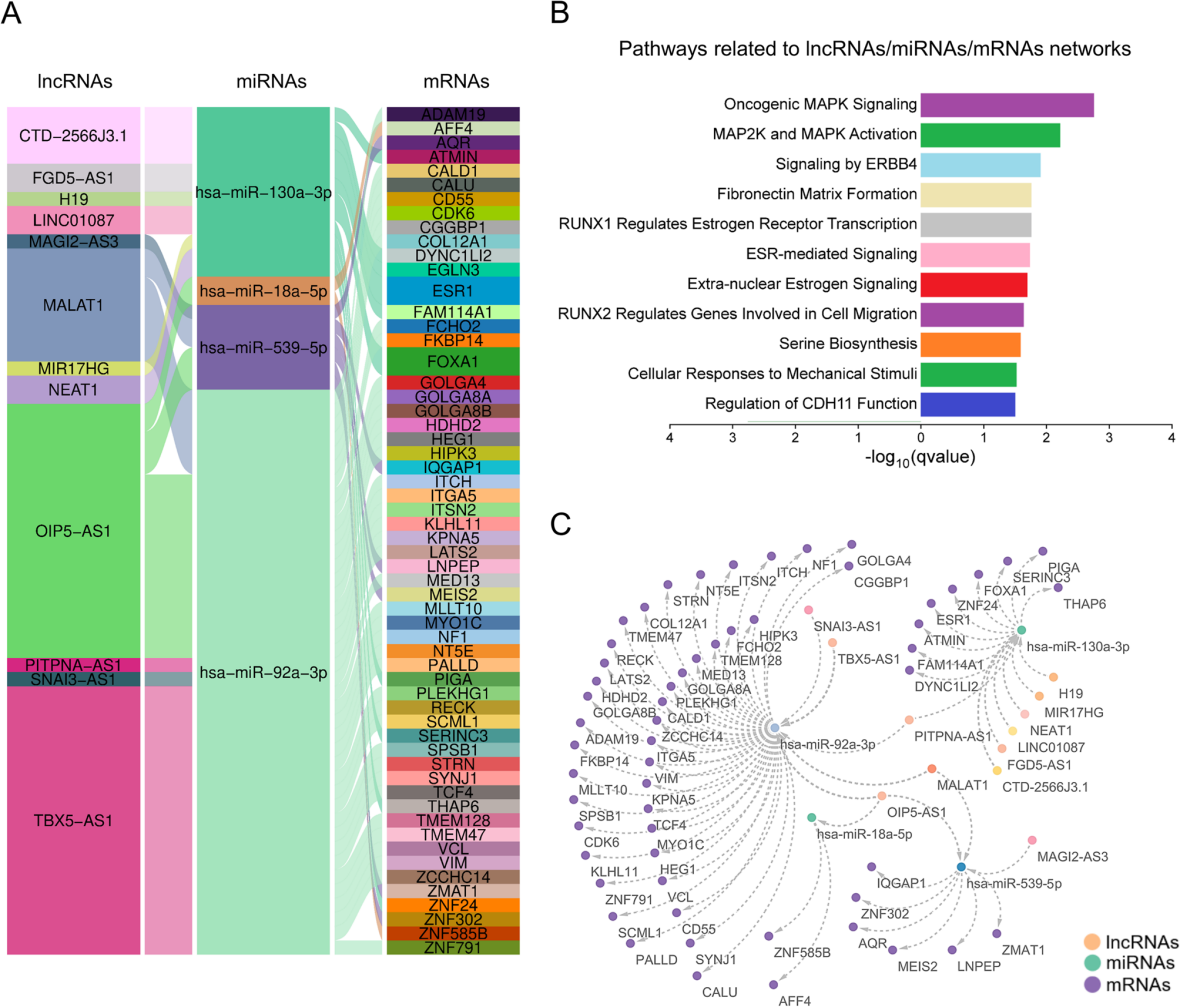
Interaction network of lncRNAs-miRNAs and mRNAs in 3D cultures of BT-474 cancer cells. **A** Interaction network between 12 lncRNAs, four downregulated miRNAs, and 58 upregulated mRNAs in 3D cultures of BT-474 cells and luminal breast tumors. The figure illustrates the predicted interactions using an alluvial diagram, with connections linking the elements. **B** Pathways enriched for interactions between lncRNAs, miRNAs, and mRNAs are ranked by their statistical significance, with the -log10(*p*-value) for each pathway shown. **C** Complex networks between the identified lncRNAs, miRNAs, and mRNAs in 3D BT-474 cultures. The nodes represent the entities (lncRNAs, miRNAs, and mRNAs), and edges illustrate their interactors

To further explore the molecular interactions within the ceRNA model, we performed an integrated analysis of miRNAs and mRNAs, focusing on miRNAs with differential expression between 3D cultures of BT-474 cells and luminal breast cancer tumors. In addition, we performed in silico prediction of long non-coding RNAs (lncRNAs) that sponge the miRNAs modulated in 3D. According to the ceRNA concept, upregulated lncRNAs can act as “sponges” for downregulated miRNAs, thereby promoting the activation of upregulated mRNAs in the network. This hypothesis is biologically plausible, as the lncRNAs predicted to interact with these miRNAs should be downregulated in expression within the ceRNA framework due to their role in sequestering miRNAs, ultimately enabling the overexpression of mRNAs that these miRNAs would usually suppress. Previously, we identified six downregulated miRNAs that were common to both the cell and tumor datasets and were selected for further analysis (Fig. 3C). Using the Encori platform, we identified 12 lncRNAs (OIP5-AS1, TBX5-AS1, MALAT1, SNAI3-AS1, PITPNA-AS1, FGD5-AS1, H19, NEAT1, MIR17HG, MAGI2-AS3, CTD-2566J3.1, and LINC01087) that were predicted to interact with four downregulated miRNAs (hsa-miR-539-3p, hsa-miR-92a-3p, hsa-miR-18a-5p, and hsa-miR-130a-3p). These miRNAs were found to regulate 58 upregulated mRNAs in 3D cultures of BT-474 cells (Fig. 4). Subsequently, we conducted an enrichment analysis of these interaction networks to identify the biological processes in which this ceRNA network may be involved. Notably, we observed potential involvement in key processes related to the tumor microenvironment of cells in 3D culture, such as oncogenic MAPK signaling, fibronectin matrix formation, estrogen receptor transcription, genes involved in cell migration, and cellular responses to mechanical stimuli (Fig. 4B). These processes suggest that the lncRNAs and miRNAs within the network may be regulating crucial aspects of tumor biology, such as MAPK signaling, cell migration, extracellular matrix formation, and responses to both hormonal and mechanical stimuli, providing a more relevant context to tumor conditions. This model not only emphasizes the critical role of miRNAs as key regulators but also underscores how interactions between lncRNAs, miRNAs, and mRNAs can contribute to cancer progression. These interactions may act as compensatory and regulatory mechanisms in 3D environments, replicating in vivo tumor conditions more accurately.

### Expression and biomarker potential of key MiRNAs in breast cancer and 3D culture models

**Fig. 5:**
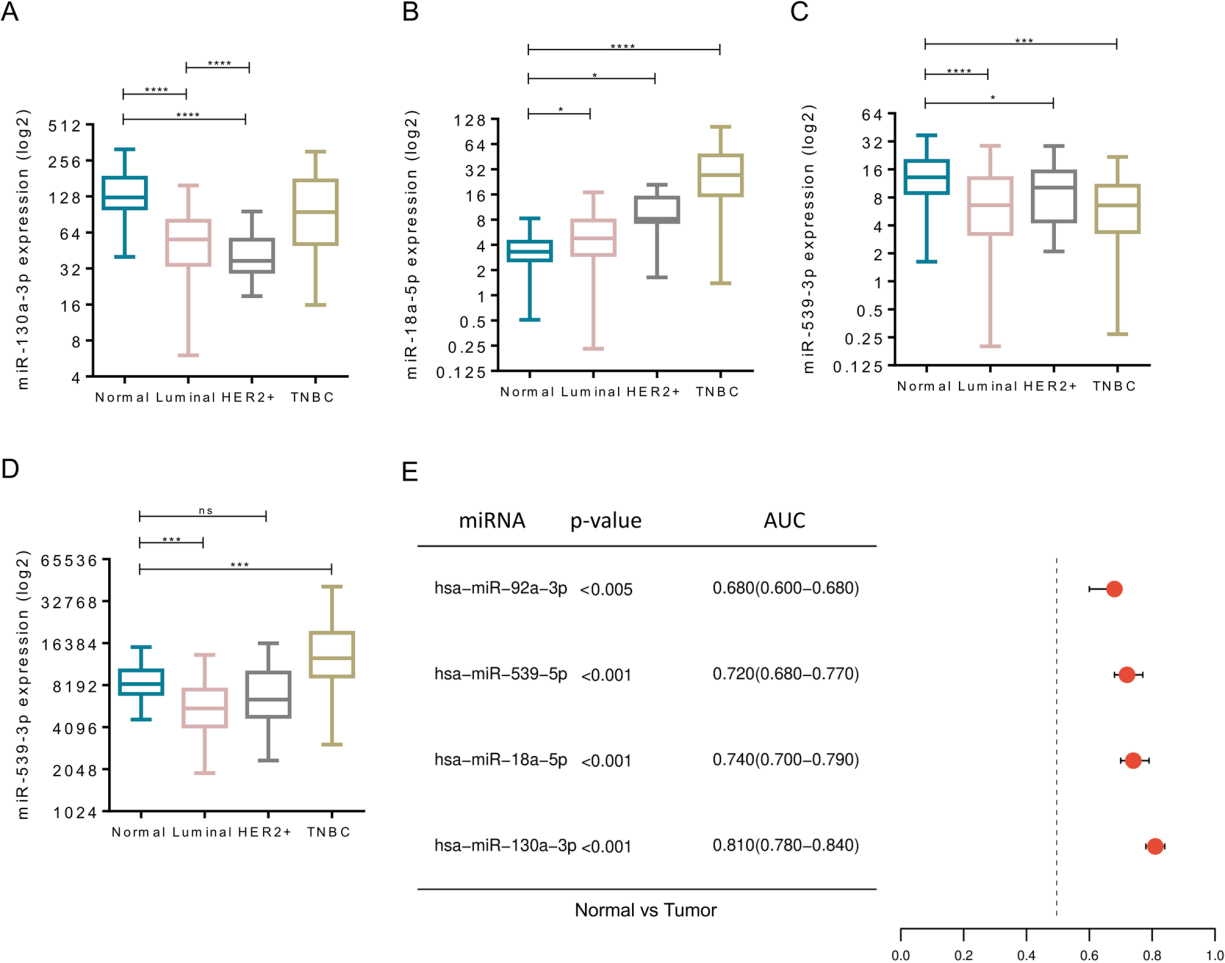
Expression Patterns of miRNAs in Breast Cancer Subtypes and Normal Tissues (**A-D**) Expression of miR-92a-3p, miR-539-5p, miR-18a-5p, and miR-130a-3p across different breast cancer subtypes (Luminal A, HER2+, TNBC) and normal tissue using TCGA-BRCA data. Data were analyzed by ANOVA followed by Tukey’s multiple comparisons test to determine statistical significance (*p ≤ 0.05, **p ≤ 0.01, ***p ≤ 0.001, ns: not significant). **E** Furthermore, the diagnostic potential of normal and breast cancer tissue was evaluated by calculating the area under the curve (AUC) values, yielding promising results: miR-92a-3p (AUC = 0.68), miR-539-5p (AUC = 0.72), miR-18a-5p (AUC = 0.74), and miR-130a-3p (AUC = 0.81). The AUC values were retrieved from CancerMIRNome

To further investigate the expression characteristics and potential prognostic value of the four miRNAs identified in the ceRNA network, we analyzed their expression patterns across different breast cancer subtypes and normal tissue from the TCGA dataset. Notably, miR-92a-3p, miR-130a-3p, and miR-539-5p were upregulated in normal tissue compared to luminal A and HER2 subtypes (Fig. 5, panels A-C). Interestingly, the expression of these miRNAs in 3D cultures of BT-474 cells was downregulated, consistent with their upregulation in normal tissue compared to tumor tissue in vivo. In contrast, miR-18a-5p exhibited higher expression levels in tumor tissue compared to normal tissue (Fig. 5D). This observation suggests that the regulatory dynamics of these miRNAs might be influenced by the tumor microenvironment, where their downregulation in 3D culture conditions may reflect their potential involvement in tumor progression. Additionally, we evaluated the diagnostic potential of these miRNAs by assessing their ability to distinguish between breast cancer and normal tissue. The area under the curve (AUC) values were promising, with miR-92a-3p showing an AUC of 0.68, miR-539-5p an AUC of 0.72, miR-18a-5p an AUC of 0.74, and miR-130a-3p exhibiting the highest AUC of 0.81 (Fig. 5E). While these results suggest that these miRNAs could serve as potential biomarkers for breast cancer, further studies would be needed to confirm their clinical utility and to understand their precise role in cancer biology. The fact that these miRNAs show significant differential expression between normal and tumor tissue, along with their distinct regulation in 3D culture, supports the hypothesis that they may play essential roles in cancer development and progression.

## Discussion

This study explores disease-relevant miRNA–mRNA–lncRNA programs by integrating our expression profiles with public datasets to build ceRNA networks. We focus on concordance between 3D cultures and luminal tumors to prioritize ceRNA axes likely active in patients rather than only in vitro, thereby increasing biological plausibility and enriching for candidates with translational potential. We highlight a ceRNA hub comprising four miRNAs (miR-92a-3p, miR-539-5p, miR-18a-5p, and miR-130a-3p) connected to 58 mRNAs and modulated by 12 lncRNAs (OIP5-AS1, TBX5-AS1, MALAT1, SNAI3-AS1, PITPNA-AS1, FGD5-AS1, H19, NEAT1, MIR17HG, MAGI2-AS3, CTD-2566J3.1, and LINC01087) (Fig. 4, panel A). This potential network, detected in both 3D cultures and luminal tumors, converges on oncogenic MAPK signaling, fibronectin-matrix organization, ER-driven transcription, migratory programs, and mechanosensitive responses, consistent with a proliferative, mechano-responsive phenotype. These observations align with prior reports showing that ceRNA networks in exosomes from luminal B breast tumors, comprising 16 lncRNAs, 15 miRNAs, and 15 mRNAs, modulate RAS–MAPK, estrogen receptor, and adhesion pathways [28]. Moreover, 3D growth of BT-474 cells has been associated with activation of MAPK signaling linked to acquired endocrine resistance [43]. About estrogen receptor signaling, data from ATAC-seq and ChIP-seq in T47D cells grown in 3D culture have indicated enrichment of chromatin open sites in the estrogen pathway, suggesting a more estrogenic program in 3D culture luminal cells [44]. This is consistent with our findings, where downregulated miRNAs facilitated the activation of estrogenic programs and MAPK signaling, enriched in our integrated miRNA-lncRNA-mRNA network analysis of 3D cultures of BT-474 cells (Fig. 4B).

In this study, we examined the expression of four specific miRNAs (miR-92a-3p, miR-130a-3p, miR-539-5p, and miR-18a-5p) across various breast cancer subtypes and normal tissue (Fig. 5, panels A-D). miR-539-5p has been identified as a potential tumor suppressor in breast cancer, with reduced expression in MDA-MB-231 and MCF7 cells and cancerous tissues. Notably, overexpression of miR-539 reduced EGFR mRNA and protein levels in these cells, and ectopic overexpression of EGFR partly reversed the inhibitory effects of miR-539-5p on cell proliferation and migration [37]. Furthermore, high levels of miR-539-5p were found to suppress epithelial-mesenchymal transition (EMT) and make cells more sensitive to cisplatin treatment [45]. These findings are consistent with our analysis, suggesting the involvement of miR-539-5p in regulating the tumor microenvironment. miR-130a-3p, often studied as a tumor suppressor, is typically downregulated in breast cancer tissues. Our analysis showed that miR-130a-3p was downregulated in 3D cultures and upregulated in normal tissue, aligning with previous studies that report a decrease in this miRNA in breast cancer samples compared to adjacent normal tissues [34]. Moreover, exosome-derived miR-130a-3p was also downregulated in breast cancer blood samples compared to healthy controls, further supporting its role as a tumor suppressor in breast cancer [46]. Both miR-92a-3p and miR-18a-5p have shown dual roles in breast cancer, where they can either promote tumor growth or inhibit it depending on the context. For miR-92a-3p, expression analysis in 2D cell cultures revealed higher levels in breast cancer cell lines (MCF7, T47D, SKBR3, MDA-MB-231) compared to the normal MCF10A cell line [47]. This miRNA is associated with increased cell proliferation by negatively regulating KLF2, a known tumor suppressor gene [47]. However, contrary to our study’s findings, miR-92a-3p expression was observed to be elevated in breast cancer tissue and serum compared to healthy controls, and was upregulated in tamoxifen-resistant cells [48]. Our findings, however, show that miR-92a-3p is downregulated in 3D cultures of BT-474 cells and overexpressed in normal breast tissue according to the TCGA database, which aligns with reports indicating high expression of miR-92a-3p in normal tissue compared to tumor areas [49]. Other in silico evaluations have also revealed significant downregulation of miR-92a-3p, with lower expression of this miRNA associated with advanced cancer stages and increased receptor expression [50]. This aligns with our enrichment analysis, which suggests that reducing miR-92a-3p enhances estrogen receptor signaling pathways. Furthermore, miR-18a-5p was downregulated in 3D cultures compared to 2D cultures and overexpressed in tumor tissue relative to normal tissue in the TCGA dataset. Previous studies have shown that miR-18a-5p is downregulated in breast cancer tissues and MCF-7 and MDA-MB-231 cells, and that its overexpression inhibits proliferation, migration, and activation of the PI3K/AKT pathway [36]. Interestingly, miR-18a-5p expression in ER + breast cancer tissues showed a negative correlation with ESR1 transcripts and ER protein [51], a finding also reflected in our enrichment analysis, which revealed the activation of this pathway. Concerning the identified lncRNAs, OIP5-AS1 was found to act as a potential sponge for miRNAs such as hsa-miR-92a-3p, hsa-miR-539-3p, and hsa-miR-18a-5p. Previous studies have reported that OIP5-AS1 is highly expressed in several breast cancer cell lines (MCF7, MDA-MB-231, ZR75, SKBR3, and MDA-MB-468) compared to the normal epithelial cell line MCF-10 A, and its overexpression can promote metastasis and invasion [52]. Similarly, other studies have shown that OIP5-AS1 is highly expressed in both clinical tumor tissues and cell lines, and knockdown of this lncRNA suppresses cell proliferation, migration, and invasion [53]. Consistent with our analysis, the interaction of OIP5-AS1 has been reported in human periodontal ligament cells by sponging miR-92a-3p [54].

Additionally, we highlighted the potential interaction between the well-characterized lncRNA MALAT1 and miRNAs hsa-miR-539-5p and miR-92a-3p. Notably, the sponge interaction of MALAT1 with miR-92a-3p has been previously described in cardiomyocytes [55]. In the context of breast cancer, specifically in BT-474 cells, MALAT1 expression is upregulated, and reducing its expression can enhance the effectiveness of trastuzumab treatment [56]. Furthermore, we identified a potential interaction between ITPNA-AS1 and miR-92a-3p, which has also been reported in gastric cancer [57]. In breast cancer, ITPNA-AS1 expression is high in triple-negative breast cancer tissues and cells, and its knockdown inhibits the viability, migration, and invasion of triple-negative breast cancer cells in vitro, as well as xenograft tumor growth in mice [58]. Another predicted interaction was between the lncRNA H19 and miR-130a-3p, which has been reported in neonatal hypoxic-ischemic encephalopathy, where H19 binds to miR-130a-3p in SH-SY5Y and N2a cells [59]. Interestingly, H19 expression was also elevated in 3D cultures of MDA-MB-231, particularly in the breast cancer stem cell population [60], suggesting that this interaction might be present and enhanced in 3D cultures of BT-474 cells. We also found a predicted interaction between NEAT1 and miR-130a-3p, previously reported in renal tubular oxidative injury [61]. NEAT1 expression is upregulated in BT474, MCF-7, MDA-MB-231, MDA-MB-453, and SK-BR-3 breast cancer cells compared to the MCF10A human breast cell line, and its high expression correlates with poor overall survival in breast cancer patients [62]. Silencing NEAT1 reduces cell proliferation, migration, and invasion, and decreases EMT in MDA-MB-453 cells. Lastly, our analysis also predicted an interaction between LINC01087 and miR-130a-3p. LINC01087 has been reported to be highly expressed in breast cancer tissues compared to tumor-adjacent tissues, and survival analysis has shown that high expression of LINC01087 is associated with poor prognosis [63]. Our study highlights the critical role of lncRNA/miRNA interactions in regulating key cancer-related processes. These interactions have been described in other pathologies, supporting the existence of such networks within the 3D culture model. Understanding these molecular mechanisms provides valuable insights into potential therapeutic targets and biomarkers for breast cancer. Further, it reinforces the utility of 3D culture systems as a more accurate model for studying tumor biology. The main limitations of our investigation are: (i) the lack of further experimental validation of the findings, which will be considered in future research, (ii) a comparative study of the changes in non-coding RNAs between the diverse subtypes of breast cancer cells, and (iii) the need for functional validation of specific deregulated lncRNA/miRNA7mRNA axes in a 3D microenvironment.

## Conclusions

This study offers valuable insights into the changes in miRNA expression under 3D culture conditions. The identification of miRNAs common between 3D cultures and luminal tumors reinforces the clinical relevance of these findings. Furthermore, this analysis underscores the importance of considering both in vitro and in vivo data when investigating cancer-related signaling networks, as it facilitates a deeper understanding of the molecular mechanisms underlying cancer progression. Through an integrated analysis of miRNAs, mRNAs, and lncRNAs, the study constructs an interaction model that could serve as a foundation for future research on biomarkers and targeted treatments. However, it is crucial to expand the experiments to validate the results in a broader context, including other cell types and animal models, to confirm the applicability of the identified miRNAs as clinical biomarkers and their therapeutic potential. Additionally, this exploratory study relies on microarray-based estimates of miRNA. Although we profiled expression in biological triplicates, analyzed TCGA data, prioritized inverse miRNA–mRNA pairs, and corroborated interactions in miRNet and ENCORI (including curated and CLIP-supported evidence: PAR-CLIP/HITS-CLIP), the reported networks should be considered a basis for future experimental validation.

## Supplementary Information

Supplementary Material 1.

## Data Availability

Microarray data that support the findings of this study have been deposited in the GEO Omnibus database with the primary accession number GSE299423.
